# Time to acute kidney injury and its predictors among newly diagnosed Type 2 diabetic patients at government hospitals in Harari Region, East Ethiopia

**DOI:** 10.1371/journal.pone.0215967

**Published:** 2019-05-02

**Authors:** Lemma Demissie Regassa, Yigzaw Kebede Gete, Fantahun Ayenew Mekonnen

**Affiliations:** 1 Department of Epidemiology and Biostatistics, Collage of Health and Medical Sciences, Haramaya University, Haramaya, Ethiopia; 2 Department of Epidemiology and Biostatistics, Institute of Public Health, Collage of Medicine and Health Sciences, University of Gondar, Gondar, Ethiopia; Istituto Di Ricerche Farmacologiche Mario Negri, ITALY

## Abstract

**Background:**

Incidence of Acute Kidney Injury (AKI) among Type 2 diabetic patients is significantly increasing. But, earlier studies has focused on the admitted patients which may hide the true nature of the Acute Kidney Injury among Type 2 Diabetic (T2D) patients. So, this study was conducted to determine the time to Acute Kidney Injury and its predictors among Type 2 Diabetic patients in Harari Region, East Ethiopia.

**Methods:**

We conducted a retrospective cohort study among type 2 diabetic patients who had been receiving treatment at government hospitals of Harari region, Ethiopia from 2013 to 2017. We extracted data from patients’ medical records. We estimated incidence rate and compared survival curves between different exposure groups using Kaplan-Meier and log-rank test. Weibull regression model was fitted to the data to identify the predictor variables. Variables with p-value <0.05 were considered statistically significant.

**Results:**

Overall, 14.5% (95%CI: 11.7–17.9) of the study population developed acute kidney injury, with median survival time of 57 months. The significant predictors were physical activity [Adjusted Time Ratio (ATR):95%CI; 0.6 (0.49–0.75)], congestive heart failure [ATR:95%CI; 0.84 (0.71–0.99)], chronic kidney disease [ATR:95%CI; 0.77(0.65–0.91)], hypertension [ATR:95%CI; 0.78(0.65–0.91)], obesity [ATR:95%CI; 0.84(0.74–0.96)], diabetic nephropathy [ATR:95%CI; 0.80(0.65–0.98)], diuretics & beta blockers [ATR:95%CI; 0.85(0.74–0.97)], and delay of follow-up [ATR:95%CI; 0.97(0.96–0.98)].

**Conclusions:**

Incidence of acute kidney injury was high in our study area. Hence, identification and controlling of comorbidities along with regular monitoring of kidney function are needed to prevent or delay the risk of acute kidney injury in type 2 diabetic patients.

## Introduction

Diabetes mellitus (DM) is a chronic metabolic disorder that occurs when the body’s response to insulin is impaired because of the incapacity of pancreas to produce enough insulin or the body cannot effectively use the insulin produced [1,2]. Type 2 Diabetes is the most common form of DM known by the body’s progressive resistance to the normal actions of insulin and/or gradual loss of the capacity to produce enough insulin in the pancreas [2,3].

The Burden of Diabetes is rising in alarming rate, becoming among the three priority non-communicable chronic disease in the World [1]. Globally, 422 million adults were living with DM in 2014, and is expected to rise above 700 million by 2030 [1,4]. The majority of adults with DM were T2D patients [1]. In Ethiopia, 2,567 (5.2%) adults were diabetic in 2015 [1,5] with annual average rise of 5.4%. The prevalence estimated from an institution based study conducted in Hossana, Ethiopia was 8.4% [6].

Acute kidney injury is the sudden and rapid increase in serum Cr (a rapid decrease in eGFR(Glomerular Filtration Rate)) [7]. It replaces terms such as acute renal failure and acute renal insufficiency, which previously have been used to describe the same clinical condition [8].

Evidence indicated that despite intensive glucose control provided by medication, patients with T2D remain at increased risk for kidney-related complications [9]. This complication is due to the damage in the microvascular and nerves of the kidney which results in fluid retention, leading to kidney injury [2].

Studies conducted at different setups and times indicated that morbidity of Acute Kidney injury has been increasing over the past few decades [8,10,11]. Studies conducted in the UK in 2012 and 2017 among T2D patients revealed an AKI incidence rate of 8.6% and 198 per 100,000 person-years, respectively [12,13]. A Study conducted in Ghana, Nigeria and Kenya reported that 13.4% of T2D patients had impaired kidney function. Acute Kidney injury is responsible to higher admission rates, long time hospital stay, risk of progression to chronic kidney disease, and increased mortality [14–16]. Currently, the incidence of AKI is about almost 20% in middle-Europe and around 32% in Africa [17].

Data on the rate of impaired kidney function and its contributing factors on patients with T2D is scarce in Ethiopia. There is little evidence on the development of AKI and its association with change in biomarkers of kidney function, specifically, in the country. Even, most of the prior studies has focused on hospitalized patients or patients in intensive care units. So, this study was conducted to determine the time to Acute Kidney Injury and its predictors among type 2 Diabetes Mellitus patients in government hospitals of Harari Region, Ethiopia. The findings will be helpful for the timely identification and treatment of AKI thereby decreasing the risks of progressive CKD (chronic kidney disease) and other complications.

## Materials and methods

### Study design

We conducted an institution based retrospective cohort study from March 28 to April 30, 2018 among patients diagnosed with Type 2 Diabetes Mellitus from January 1, 2013 to December 31, 2017.

### Study setting

The study was conducted at three urban government hospitals in Harari Regional State, Ethiopia that have Diabetes follow-up services. The Harari Regional State is one of the nine ethnically-based regional states of Ethiopia. Its capital is Harar, 500 km far from the capital of Ethiopia, Addis Ababa. The total population of the region by 2017 was 246,000 [18]. In the town of Harar, there are 8 Health Centers, 24 Health Posts, 7 Hospitals (2 private, 3 Federal, 1 non-governmental organization and 1 regional state owned). This study included 3 government hospitals. These hospitals were Hiwot Fana specialized referral and teaching hospital, Jugal general hospital, and Federal Police hospital.

### Study participants

The study included all patients who were newly diagnosed with Type 2 diabetes during the follow-up visits from January 1, 2013, to December 31, 2017. Patients with end-stage renal disease, renal transplant and history of dialysis at the diagnosis of Type 2 Diabetes, pregnant women, initially present with AKI or no baseline records were excluded.

### Operational definitions

**Newly Diagnosed Type 2 Diabetic (T2D) Patients**–Patients who were diagnosed for Type 2 Diabetes after December 31, 2012.

**Acute Kidney Injury–**Is an abrupt decrease in kidney function characterized by increase in Serum Creatinine (SCr) by = 0.3 mg/dl (= 26.5 μmol/l) versus the baseline or increase in SCr to 1.5 to 2 times baseline value [19,20].

**Time to acute kidney injury**- It was the time between the times of diagnosed of T2D to the development of first episode of acute kidney injury.

**Event–**Was development of AKI.

**Censored-** Patients who were not experiencing AKI until the end of the study or died or lost to follow up before experiencing AKI within the study period.

**Obese-** Patients whose baseline body mass index exceeded 30 kg/m^2^.

**Physically active-** Patients who reported doing exercise for at least 30 minute per day or working in the field.

### Sampling technique and sample size determination

The sample size was calculated using the powerSurvEpi package of R software after considering a Cox proportional hazard model assumption. Hence, at 95% Confidence level, 80% Power and 10% contingency, the final sample size determined for this study was 519 T2D patients.

### Measurement of variables

The outcome of interest for this study was time to the first Acute Kidney Injury episode in T2D patients. The explanatory variables included, the sociodemographic factors, behavioral factors, clinical factors and comorbidities.

### Data collection

We used secondary data, including patient intake form, follow up card and DM registration book, as well as the electronic information databases, recorded routinely by the hospitals for follow up, monitoring and evaluation purposes. Data were collected using checklists by health officers and nurses working in the respective hospitals. To ensure the quality of data, the filled sheet was checked for completeness and consistency by study supervisors and the principal investigator. We also cross-checked the data entry and clarified any missing data.

### Data analysis

**Fig 1:**
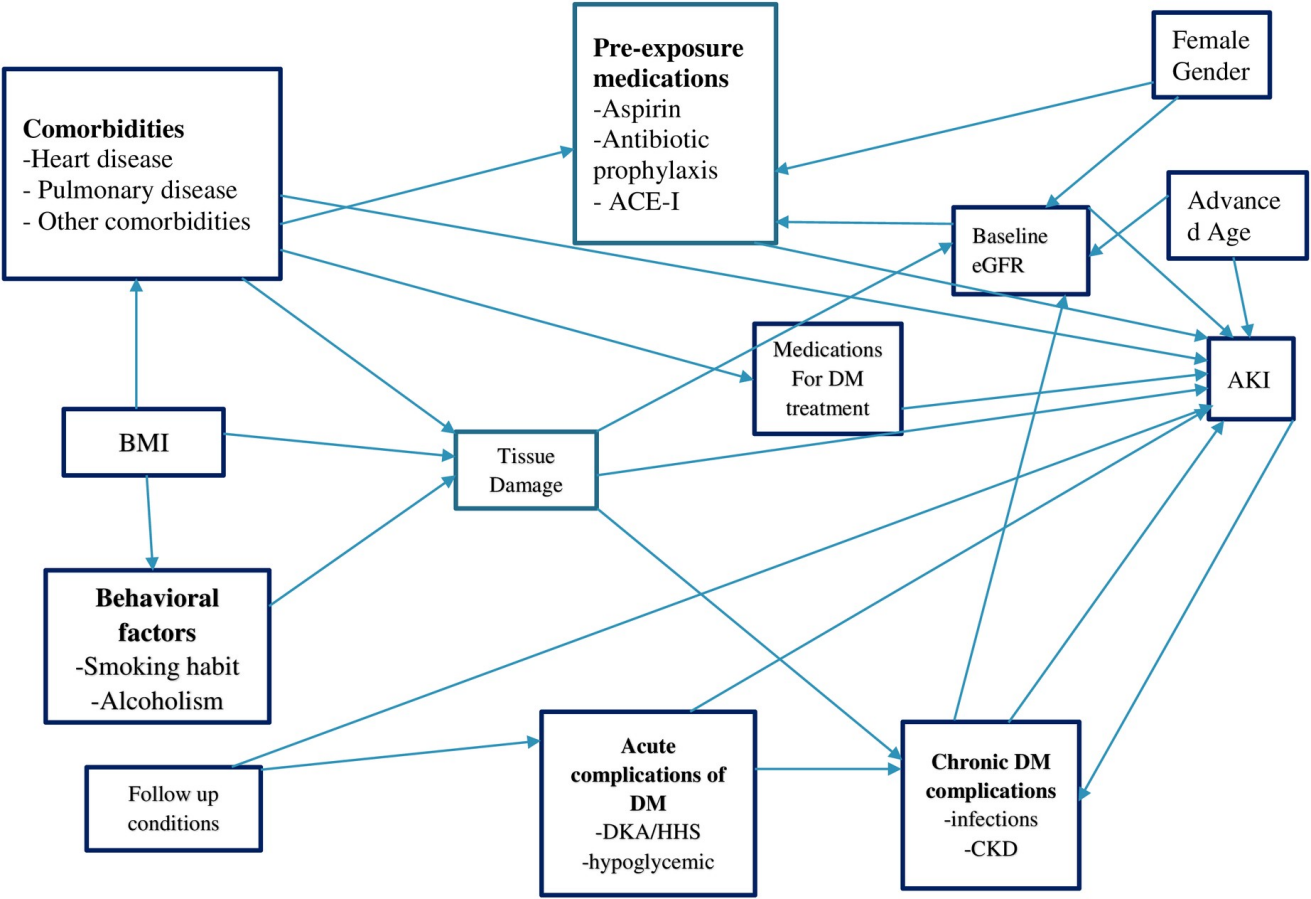
Direct acyclic graph for the risk factors of AKI among type 2 diabetes patients (Source: (Kasper 2005, Khwaja 2012, Lameire, Bagga et al. 2013, Lee 2015).

Data were entered into Epi Info Version 7. Continuous variables were described in terms of mean/median value and 25 & 75th percentiles, and categorical characteristics, including the outcome of the study were described in terms of percentages and frequencies. The incidence rate of acute kidney injury was also calculated for the entire cohort by dividing the total number of incident cases of AKI to the total person-years of follow-up. We estimated the median survival time and compared it across groups of key characteristics using survival curves and Kaplan- Meier and log-rank test. Parametric distribution for baseline hazard function was assumed after suggested by graphical diagnosis. Based on the Akaike Information Criteria (AIC) comparison, the Weibull regression model was identified to be an efficient model. Using this model we first examined bi-variable associations between the predictors and outcome for all variables. Potential predictors adopted from the previous studies were initially selected by using the direct acyclic graph (DAG) (Fig 1).

The full-information model was tested by including all available predictor variables using a backward-entry stepwise procedure with a threshold of P<0.20. Finally, model fitness was checked using Schoenfeld residuals to test for proportionality, Cox-Snell residuals to test for goodness of fit, martingale residuals to look for non-linearity and dfbeta for influence, and appropriateness of a Weibull model was checked by plotting the log negative log of the Kaplan–Meier survival estimates against the log of time. The risk estimates were expressed as time ratios and all statistical tests were done at α = 0.05 level of significance. The analysis was performed by R statistical software version 3.5.0.

### Ethical considerations

The study protocol was reviewed and approved by the Ethical Review Committee of the College of Medicine and health sciences, University of Gondar. Letter of cooperation was obtained from the respective Hospital Directors after submitting the proposal. For the sake of privacy and confidentiality, no personal identifiers such as names, address and any private information were collected. Data were handled confidentially during all phases of research activities using anonymous medical registration numbers as an identification. Soft copy registrations were protected using a password.

## Results

We included 502 T2D patients who met the inclusion criteria of which 254 (50.6%) were from Hiwot Fana specialized and teaching hospital, 129 (25.7%) from Jugal hospital and 119 (23.7%) from Federal Police hospital. The age of participants at baseline ranged from 12 to 86 years with the mean of 48.31± 14.85 (95% CI: 33.46–63.22) years. Females constituted 57.2% of the patients. Participants who were married accounted for 84.9% (436/ 502) and 193 (38.4%) participants were government employees. The majority, 368 (74.1%) of the participants were urban dwellers (Table 1).

**Table 1:** Socio-demographic characteristics of T2D patients at government hospitals in East Ethiopia from 2013 to 2017.

Characteristics	Frequency	Percent (%)
Place of follow up		
Hiwot Fana Hospital	254	50.6
Jugal Hospital	129	25.7
Police Hospital	119	23.7
Sex		
Male	215	42.8
Female	287	57.2
Marital Status		
Married	424	84.4
Widowed	39	7.8
Others	39	7.8
Residence		
Urban	368	74.2
Rural	72	14.5
Semi Urban	56	11.3
Occupation		
Gov't Employee	193	38.4
Private Work	126	25.1
Unemployed	70	13.9
Farmer	48	9.6
Unspecified	35	7.0
Retired	30	6.0

Occasional physical activity was reported by 179 (36.2%) of the participants. A previous history of smoking was reported by 13 (2.6%) patients of whom 17 (3.4%) reported smoking an average of halve a package of cigarettes per day. History of ever drinking alcohol was reported by 36 (7.2%) patients. Family history of Diabetes was reported by 119 (23.7%) patients. The mean baseline serum creatinine was 1.04 (0.59SD) mg/dl while the mean eGFR was 91.56 (80.9SD). The body mass index of the participants was within the range of 23.2–27.5 kg/m^2^ and the mean fasting blood glucose level was 215.67 (118.9SD) mg/dl.

Regarding the co-morbidities that were identified, a total of 201 (40%) had a history of hypertension, 30.1% experienced different types of infections in the last four years.

Regarding the management of DM, 441 (87.8%) were on oral medication and the remaining were treated with glucose injection. From the total patients on oral medications, 246(49%) were taking both metformin and Glibenclamide, the remainders were on metformin (Table 2).

**Table 2:** Comorbidity among T2D patients at government hospitals in East Ethiopia from 2013 to 2017.

Variables (%)	Censored	AKI
Oral Anti-diabetes	376 (85.3)	65 (14.7)
Family history	98 (82.4)	21 (17.6)
History of diabetic ketoacidosis	68 (85.0)	12 (15.0)
CHF	12 (52.2)	11 (47.8)
CKD	15 (55.6)	12 (44.4)
HTN	139 (69.2)	62 (30.8)
Obesity	43 (63.2)	25 (36.8)
Foot Ulcer	17 (58.6)	12 (41.4)
Retinopathy	27 (65.9)	14 (34.1)
Diabetic nephropathy	18 (78.3)	5 (21.7)
Infection	123 (81.5)	28 (18.5)
Take Non-steroidal anti- inflammatory drugs	139 (79.0)	37 (21.0)
Diuretics or beta blockers	131 (74.0)	46 (26.0)
Take Anti-biotics	123 (83.1)	45 (16.9)
Total	429 (85.5)	73 (14.5)

**Fig 2:**
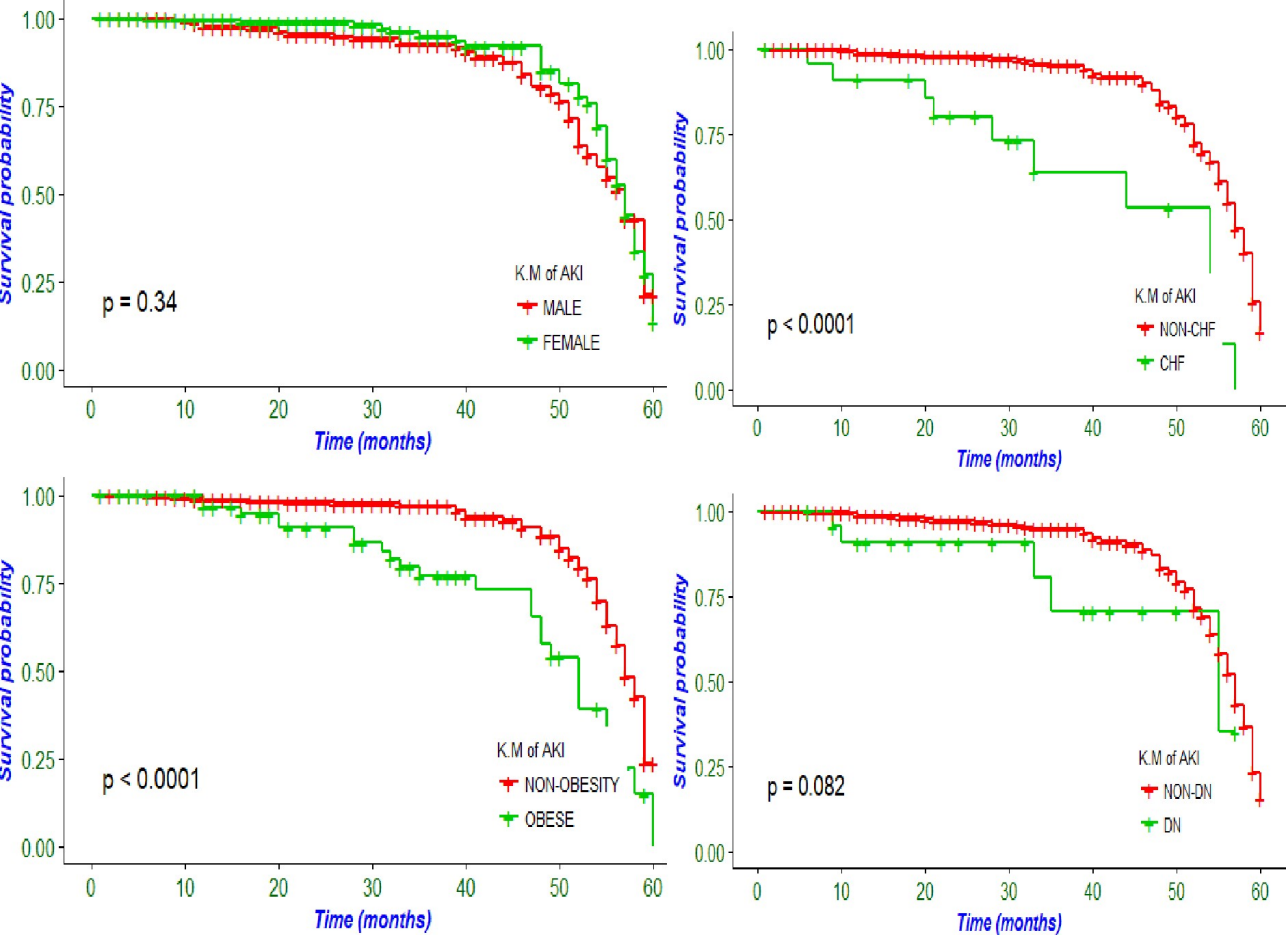
AKI incidence by selected variables among T2D patients in East Ethiopia, from 2013 to 2017.

During the follow up, a total of 73 patients developed AKI. The cumulative incidence of acute kidney injury was 14.5% (95%CI; 11.7–17.9). The incidence density was 6 per 100 patients per year and the median survival time of patients to experience the first episode of AKI was 57 (QR±2) months (Fig 2).

In the multivariable analysis eight variables, which included physical activity [ATR:95%CI; 0.61 (0.49–0.75)], congestive heart failure [ATR:95%CI; 0.84 (0.71–0.99)], Chronic kidney disease [ATR:95%CI; 0.77 (0.65–0.91)], Hypertension [ATR:95%CI; 0.78 (0.65–0.91)], Obesity [ATR:95%CI; 0.84 (0.74–0.96)], Diabetic nephropathy [ATR:95%CI; 0.80 (0.65–0.98)], Diuretics & Beta Blockers [ATR: 95%CI; 0.85 (0.74–0.97)], and Delay of follow up [ATR:95%CI; 0.97 (0.96–0.98)] were statistically significantly associated with AKI among T2D patients (Table 3).

**Table 3:** Multivariable regression among T2D patients at government hospitals in East Ethiopia, from 2013 to 2017.

Variables	Non-AKI	AKI	CTR	ATR (95%)
Mean age (years)	47.22	54.76	1.02 (1.01, 1.04)	0.99 (0.99, 1.00)
Sex				
**Female**	249	38	0.91(0.57, 1.45)	1.03 (0.92, 1.16)
**Mal**	180	35	11	11
Physical Activity				
**Inactive**	65	66	0.48(0.37, 0.63)	0.61 (0.49, 0.75)
**Active**	364	7	11	1
Smoking Habit				
**Current smoker**	7	6	0.82 (0.67, 0.99)	0.99 (0.81, 1.22)
**Past smoker**	12	5	0.80 (0.65, 0.99)	0.92 (0.72, 1.17)
**Non-smoker**	404	61	1	1
Alcoholism				
**Yes**	24	12	0.79 (0.68, 0.91)	0.88 (0.75, 1.03)
**No**	405	61	1	1
Congestive Heart Failure				
**Yes**	12	11	0.73 (0.62, 0.85)	0.84 (0.71, 0.99)
**No**	417	62	1	1
Chronic Kidney Disease				
**Yes**	15	12	0.74 (0.63, 0.86)	0.77 (0.65, 0.91)
**No**	414	61	1	1
Obesity				
**Yes**	43	25	0.78 (0.69, 0.89)	0.84 (0.74, 0.96)
**No**	386	48	1	1
Foot Ulcer				
**Yes**	17	12	0.84 (0.73, 0.98)	1.03 (0.87, 1.22)
**No**	412	61	1	1
Antibiotics History				
**Yes**	123	45	0.84 (0.74, 0.95)	0.97 (0.86, 1.10)
**No**	306	28	1	1
Anti HTN				
Non-diuretics Diuretics/BB	298 131	27 46	0.84 (0.74, 0.94) 1	1.18 (1.03, 1.35) 1
Mean Baseline BMI (kg/m^2^)	23.46	25.34	0.99 (0.96,1.01)	1.01 (0.99, 1.03)
Mean Delay (years)	0.88	2.07	0.98 (0.97, 0.98)	0.97 (0.96, 0.98)
Glibenclamide				
**Yes**	205	41	1.04 (0.93, 1.16)	0.97 (0.85, 1.11)
**No**	224	32	1	1
Diabetic Nephropathy				
**Yes**	18	5	0.96 (0.86, 1.07)	0.80 (0.65, 0.98)
**No**	411	68	1	1
History of Infection				
**Yes**	123	28	0.85 (0.68, 1.06)	1.08 (0.94, 1.24)
**No**	306	45	1	1
Hypertension				
**Yes**	297	62	0.93 (0.83,1.04)	0.78 (0.65,0.94)
**No**	132	11	1	1

## Discussion

This study has examined the time to develop acute kidney injury and its determinant factors in Type 2 Diabetic patients. A total of eight factors were identified to determine the time to develop AKI in T2D patients. These factors included, physically activity, hypertension, chronic kidney disease, congestive heart failure, diabetic nephropathy, diuretic anti-hypertension medication, delay to start DM follow-up and obesity.

Our study revealed that the incidence of AKI was 14.5% (95%CI; 11.7–17.9) in T2D patients. This is in line with a study conducted in Kenya, Ghana and Nigeria that around 13.4% of T2D patients have experienced Kidney injury during the follow up in these countries [21]. However, it is higher than the findings of two cohort studies conducted in the UK [12,13]. The reason for the elevation of AKI in the current study and a study in Kenya, Ghana and Nigeria as compared to the UK might be due to late diagnosis and initiation of follow up of diabetic patients and lack of strict monitoring of complications in those African countries including Ethiopia. It might also be due to contextual differences present between developing and developed nations.

In our study, diabetic nephropathy was independently associated with AKI. The time to develop AKI was significantly faster among the T2D patients with diabetic nephropathy compared to T2D patients without diabetic nephropathy. This is consistent with the result of a review study in Africa and a study conducted in New Zealand [22,23].

In this study, the risk of developing AKI among hypertensive patients was higher by three-fold than non-hypertensives. Several studies from different settings supported this result. For example, a study conducted from 28 countries indicated T2D patients with hypertension were at increased risk of developing AKI [24]. Another study conducted to assess the impact of T2D on kidney function in Sub Saharan Africa has also suggested that hypertension was positively associated with the AKI [21]. There are also review studies concluding that hypertensive individuals had a higher risk of AKI than those without hypertension (HTN) [17,25]. This might be because of total peripheral blood vessels resistance, a rise in extracellular fluid volume, and nephron & tubular necrosis [26].

Obesity was found to accelerate the time to AKI irrespective of its severity in this study. After controlling for the other factors, the time to develop the AKI among obese patients was significantly decreased compared to non-obese patients. This result was in line with the study conducted in the UK in 2016, which reported that obesity was the risk factor in the presence or absence of HTN, Congestive heart failure (CHF) or other cardiac comorbidities [13]. A similar single center study in 2016 on critically ill patients admitted to the intensive care unit also identified obesity as an independent predictor of AKI [27]. The unfavorable effect of obesity might be the result of the macula densa feedback mechanism and indirectly by initiating other chronic comorbidities or it might be due to the collective result of the increased glomerular wall tension and glomerular hypertrophy caused by the increased renal tubular sodium reabsorption and renal lipotoxicity [28,29].

Congestive heart failure and chronic kidney disease were significant predictors of AKI. Congestive heart failure patients, irrespective of underline cause, were at higher risk of developing AKI than those who did not experienced CHF. A study conducted in the UK, which analyzed the effects of specific co‐morbidities with T2D found that CHF has increased the risk of AKI [13]. There are also other studies supporting this finding [24,25,27,30].

This study has also identified that nephrotoxic drugs were important predictors of AKI. This is similar a study conducted in the UK and from other 28 countries [13,24].

Delay for follow up after the patients were diagnosed with Type 2 Diabetes was independent predictor of AKI development. The time to AKI development has decreased significantly among patients delayed at least for a month to start treatment. Previous studies also suggested that the early initiation of follow up treatment after the diagnosis is a crucial step to prevent or delay the development of complications from Type 2 diabetes. Even though the recommended visit is varied, it is advised that patients should have a comprehensive assessment with intensive follow-up. Early initiation of follow up is helpful in the control of hyperglycemia, which is the initial cause of varies long-term complication in T2D patients [31,32].

This study is not without limitations. The exact time of onset of T2D and AKI was not known. As a result, it was difficult to determine how long an individual delayed to start medication. This result might have affected by residual confounding since some data were missed from the record. Important investigation for kidney trajectories and HbA1c were not available for all patients. Despite, of these limitations, this study assessed the factors that determine the time to develop acute kidney injury using the best possible model available.

The incidence of acute kidney injury was relatively high among the Type 2 Diabetic patients. It was confirmed that acute kidney injury among Type 2 Diabetic patients was determined by physical activity, congestive heart failure, chronic kidney disease, hypertension, Obesity, Diabetic nephropathy, Diuretics & Beta Blockers and Delay of follow up.

We suggest that starting the follow up immediately after diagnoses, a regular checkup for comorbidities, and promoting physical activity are advisable to reduce or delay the risk of AKI.

## Supporting information

S1 Data This is the data set of a study of AKI incidence and its associated factors among Type 2 Diabetic patients. (XLSX)
